# Combination Therapy for Sustainable Fish Oil Products: Improving Cognitive Function with n-3 PUFA and Natural Ingredients

**DOI:** 10.3390/biomedicines12061237

**Published:** 2024-06-03

**Authors:** Anthony Arsecularatne, Rotina Kapini, Yang Liu, Dennis Chang, Gerald Münch, Xian Zhou

**Affiliations:** 1 NICM Health Research Institute, Western Sydney University, Westmead, NSW 2145, Australia; 20205690@student.westernsydney.edu.au (A.A.); 20549389@student.westernsydney.edu.au (R.K.); d.chang@westernsydney.edu.au (D.C.); g.muench@westernsydney.edu.au (G.M.); 2 School of Medicine, Western Sydney University, Campbelltown, NSW 2560, Australia; 3 School of Science, Western Sydney University, Paramatta, NSW 2150, Australia

**Keywords:** fish oil, natural products, synergy, cognitive function, neuroinflammation, oxidative stress

## Abstract

Long-chain polyunsaturated omega-3 fatty acids (n-3 PUFAs), particularly docosahexaenoic acid (DHA) and eicosapentaenoic acid (EPA), are recommended as beneficial dietary supplements for enhancing cognitive function. Although fish oil (FO) is renowned for its abundant n-3 PUFA content, combining FO with other natural products is considered as a viable option to support the sustainable development of FO products. This review aims to provide comprehensive insights into the advanced effects of combining FO or its components of DHA and EPA with natural products on protecting cognitive function. In two double-blind random control trials, no advanced effects were observed for adding curcumin to FO on cerebral function protection. However, 16 week’s treatment of FO combined with vitamin E did not yield any advanced effects in cognitive factor scores. Several preclinical studies have demonstrated that combinations of FO with natural products can exhibit advanced effects in addressing pathological components in cognitive impairment, including neuroinflammation, oxidative stress, and neuronal survival. In conclusion, evidence from clinical trials for beneficial use of FO and natural ingredients combination is lacking. Greater cohesion is needed between preclinical and clinical data to substantiate the efficacy of FO and natural product combinations in preventing or slowing the progression of cognitive decline.

## 1. Introduction

With the increasing human life span, there is a growing interest in maintaining a healthy later life, particularly concerning cognitive function [1]. Projections have forecasted a significant rise in the number of individuals experiencing dementia, with estimates suggesting a doubling every two decades, reaching 42 million in 2020 and a projected 81 million in 2040 [2]. Clinical dementia is diagnosed when cognitive loss becomes severe enough to impact daily life [3]. However, before progressing into clinical dementia, mild cognitive impairment (MCI) is considered as an early stage, estimated to have over a 50% chance of progressing to clinical dementia within four years [4]. While viable treatments for dementia are limited, any effective intervention that could help to prevent early-stage cognitive decline from developing into dementia is valuable.

Long-chain polyunsaturated omega-3 fatty acids (n-3 PUFAs), primarily docosahexaenoic acid (DHA) and eicosapentaenoic acid (EPA), have shown various health benefits throughout the life span [5,6,7]. As part of a healthy diet, DHA and EPA can improve brain function [8], regulate blood pressure and lipid metabolism [9], exhibit anti-inflammatory [10] and anti-tumour effects [11], protect against nephrotoxicity, and improve exercise training and performance [12]. In particular, there has been a continuous interest in n-3 PUFAs to improve cognitive function in children and prevent cognitive decline and memory loss in older people [13,14,15]. A systematic review and meta-analysis consisting of 34 studies with 12,999 recruited participants (1031 infants, 1517 children, 3657 adults, and 6794 elderly individuals) revealed that n-3 PUFA supplements significantly improved cognitive development in infants, including the mental development index, language, motor, and cognitive function. Interestingly, n-3 PUFAs seemed ineffective in enhancing cognitive function across the composite memory, executive function, and processing speed domains in children, adults, and older people, except for the attention domain [16]. One potential explanation for this finding could be the presence of underlying psychiatric disorders, such as attention-deficit disorders and depression, within the cohorts of children and adults. In addition, the effectiveness of n-3 PUFAs in treating Alzheimer’s disease (AD) or associated neurodegenerative diseases has not been demonstrated [17,18,19]. Nevertheless, systematic reviews and meta-analyses have suggested that the incorporation of n-3 fatty acids (FAs) into the diet should be encouraged, supported by preliminary evidence of benefits for cognitive and memory functions in non-demented adults [20] or those with MCI in randomized controlled trials (RCTs) [21,22].

Fish oil (FO) contains a rich amount of n-3 PUFAs, serving as the primary source for the human diet. FO has been postulated to have multiple modalities through which it exerts biological activity in the brain, including neuronal membrane fluidity, gene expression, and modulating the release of pro-inflammatory molecules [23]. Neuronal membrane fluidity is critical to cognition, as higher cholesterol levels can decrease membrane fluidity, reducing the ability of a cell to respond to signalling events. FO is believed to reduce cholesterol in the neuronal membrane, a phenomenon associated with aging and neurodegenerative disease [24]. In addition, FO has been found to reduce the expression of pro-inflammatory genes, which results in an improved microvascular environment for neuronal survival and function [25].

The concentrations of n-3 PUFAs vary among species and between fish that are farm-raised and those caught in the wild. While crucial for supplying n-3 PUFAs through direct human consumption, fish, and, consequently, omega-3, can also be utilized as feed for aquaculture or terrestrial animal production, either in whole form or as fishmeal and FO. This practice often results in indirect and potentially inefficient pathways to human consumption. Moreover, with fish catch being plateaued, the global supply from traditional sources of omega-3 is becoming insufficient to meet the increasing human nutritional demands [26,27]. To promote the sustainable development of omega-3 nutraceuticals, vegetable-oil-derived omega-3 is primarily considered as an alternative source, although it is not efficient in providing EPA as yet [27]. Research on microbial EPA/DHA production using genetically modified organism technology is underway for future omega-3 supply; however, it appears to be an expensive option [27].

To promote the sustainable development of n-3 PUFAs, exploring combination therapy involving FO and other natural products is worth considering. Numerous studies have suggested that well-designed combination therapies can enhance efficacy while reducing the dosage needed for each component to address complex pathological conditions, such as cognitive decline [28,29,30,31]. In this context, the substitution of a portion of FO with other nutraceuticals, while maintaining or even improving efficacy in promoting cognitive function, is worth investigation.

This study aims to comprehensively review existing evidence on combination therapies involving FO and its beneficial components of DHA and EPA with other natural products and determine if such combinations offer additional benefits. Additionally, this review seeks to deepen the understanding of the mechanisms behind synergistic interaction by addressing the key pathological components involved in cognitive function. The goal is to gain new insights for the development of optimized FO combination therapies for cognition, thereby contributing to sustainable development.

## 2. Methods

Journal articles were researched in Google Scholar, PubMed, Science Direct, and Wiley Online Library relating to the combined synergy of fish oil and natural products used together to support cognitive function.

The search terms consisted of four components. The MeSH search terms related to fish oil include: “fish oil”, “Omega 3”, “Eicosapentaenoic acid” or “EPA”, and “Docosahexaenoic acid” or “DHA”.

The search terms related to natural products included: “herbal”, “herbal compounds”, “herbal extracts”, “herbal medicine”, “herbal formulation”, “herbal ingredients”, “natural compounds”, “plants”, “medicinal plant”, “Chinese herbal medicine” “Chinese herbal formula”, and “Chinese herbal formulations”.

The search terms related to cognition included: “neurodegenerative disorders”, “cognition dysfunction”, “cognition decline”, and “neuroinflammation”.

The synergy search terms were enhanced by synonyms such as: “synergistic”, “synergism”, “enhance”, “promote”, “combine”, “combination”, “formulate”, “formulation”, “compatible”, and “interaction”.

The articles included in the review (1) implied the synergy or advanced activity of combined fish oil and natural compound(s) with quantitative analysis, (2) statistical comparison of the combined effects to the individual effect(s), and (3) defined the interaction (synergistic/antagonistic) for the combined therapy. Excluded articles were those that (1) lacked a comparison of the synergy or pharmacological activity between the combined to monotherapy activities, (2) contained no natural ingredients in the combined therapy with fish oil, (3) used unspecified dosages for both combined or monotherapies, (4) systematic reviews or meta-analyses, and (5) not relevant to cognitive function.

The literature search was conducted by Y.L., A.A., and R.K. independently and was reviewed by X.Z.

## 3. Study Selection

**Figure 1:**
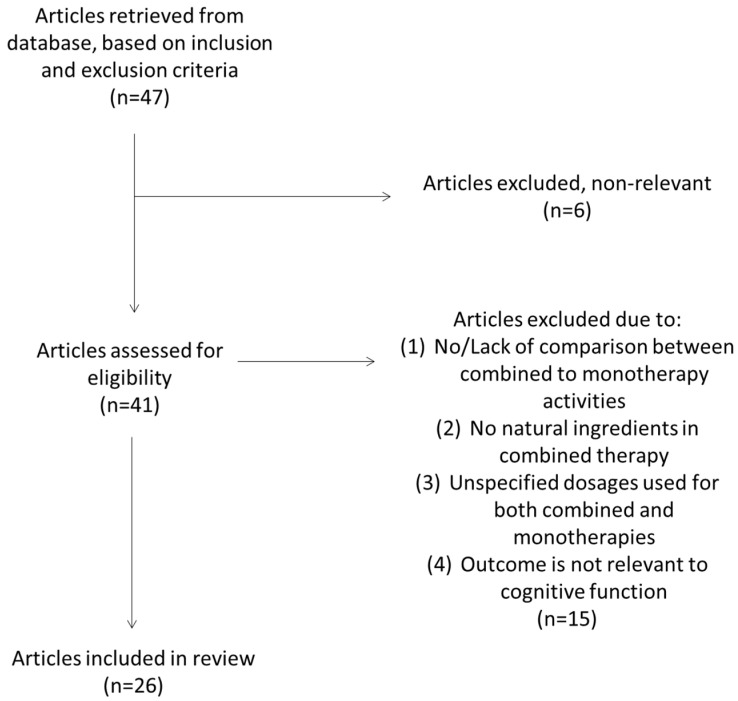
Flow diagram of retrieved journal articles.

A total of 47 studies were collected from our search strategy and 41 were included based on their relevance. Six studies were excluded during screening that were either systematic reviews, meta-analyses, or not relevant to cognition or neuroinflammation activity. The remaining articles were assessed for their eligibility and 15 articles were excluded due to the exclusion criteria. The method of the search strategy and retrieved articles are shown in Figure 1. Finally, 26 papers were included for our review. 

## 4. Clinical Evidence on Combinations of FO and Natural Products in Improving Cognition

A systematic review suggested that the ingestion of n-3 PUFAs increased learning, memory, cognitive well-being, and blood flow in the brain based on 42 reviewed RCTs [21]. However, a DHA-enriched diet only showed benefits in the early stages of cognitive decline rather than AD [32]. Welty (2023) demonstrated that the clinical outcomes of FO supplements on cognitively health individuals were not conclusive among 15 RCTs, potentially due to varied study dosages, trial durations, and characteristic factors of individuals [32]. The effectiveness of combining FO/DHA with other nutraceuticals was explored in seven studies to investigate whether the addition of nutraceuticals could provide greater benefits in protecting cerebral function and improving cognitive function.

Curcumin is the major bioactive compound from the stem of *Curcuma longa* L., a member of the *Zingiberaceae* family. Curcumin is a valuable natural product with prominent antioxidant [33,34], anti-microbial [35], anti-inflammatory [36], and anti-depressant properties [37]. Two double-blind RCTs investigated if adding curcumin (160 mg/d, Blackmores Brain Active^TM^, Blackmores Institute, Sydney, Australia) to FO (2000 mg/d DHA + 400 mg/d EPA, Blackmores Omega Brain^TM^, Blackmores Institute, Sydney, Australia) for 16 weeks would yield more benefits in improving cognitive performance and cerebrovascular function than FO alone in older sedentary overweight/obese adults [38,39]. Their results suggested that each individual treatment of FO or curcumin was able to improve cerebrovascular responsiveness compared with the placebo group, but in males only. However, combining curcumin and FO did not generate any further improvement in the outcomes in relation to cognition.

Vitamin E is a fat-soluble vitamin that has several forms, including α-, β-, γ-, δ-tocopherol and α-, β-, γ-, and δ-tocotrienol. These forms are present in plant-based foods such as oils and seeds or in fortified foods, mostly in the form of α-tocopherol [40,41]. Vitamin E is known to have strong antioxidant properties [42,43,44]. In a double-blind RCT, FO (480 mg EPA + 480 mg DHA) with or without vitamin E (50 IU) was combined and orally administered daily for 16 weeks to 160 healthy male and female volunteers aged from 50 to 70 years. Their results showed that neither individual nor combined treatment resulted in any difference in their cognitive factor scores at week 16. However, there was a significant time by treatment effect for short-term memory following 6 weeks of treatment, although the placebo group performed even better [45]. This negative outcome may be attributed to the subjects selected in the study who were all healthy. Interestingly, this study also suggested that the improved spatial working memory was positively correlated with an increased plasma omega-3/6 ratio.

The term ‘B vitamins’ refers to a group of eight water-soluble vitamins that have critical roles in anabolic and catabolic metabolism. Vitamin B6, consisting of three chemically distinct compounds (pyridoxine, pyridoxal, and pyridoxamine), is crucial for regulating mental function and is involved in the remethylation of homocysteine. Three clinical trials investigated the potential correlation between the effectiveness of vitamin B supplements against MCI and plasma DHA/EPA levels to identify the most beneficial cohort. A retrospective analysis investigated the effect of daily high-dose oral administrations of vitamin B supplementation (0.8 mg folic acid, 0.5 mg vitamin B12, and 20 mg vitamin B6) on brain atrophy in elderly people with MCI. Their results showed that the B vitamin group lowered the mean atrophy rate by 40% in subjects with high plasma omega-3 fatty acid concentrations compared with that of the placebo and the subjects with low baseline omega-3 fatty acids and homocysteine levels. This study highlighted the importance of identifying suitable subgroups based on baseline plasma DHA concentrations for potential benefits [46]. Subsequently, in the identified subgroup of elderly individuals with MCI and high baseline omega-3 fatty acids (515.98 ± 235.01 serum DHA + EPA), 33% of individuals receiving high-dose B vitamins (composition not specified) were prone to developing dementia, as assessed by the clinical dementia rating scale, compared to 59% of those on the placebo. An interaction appeared to occur between B vitamins and DHA only, with EPA demonstrating a comparatively lower impact [47]. A post hoc analysis study further investigated the effect of vitamin B (500 µg V12 and 400 µg folic acid) on global cognitive functioning in elderly individuals (over 65 years old) with elevated homocysteine levels and the potential correlation with plasma DHA/EPA. Their study suggested a positive outcome when subjects were administered vitamin B over time compared to the placebo, regardless of plasma omega-3 fatty acid levels. Interestingly, when assessing DHA/EPA separately, the results demonstrated a significant interaction between B-vitamins and DHA status, with the participants benefiting significantly more from B-vitamin if they were also in the high DHA tertile. However, such a trend was not observed in measuring domain-specific cognitive functioning [48]. Whether there was any interaction in reducing homocysteine levels for combined therapy remains unknown.

Lipoic acid (LA) is a naturally occurring dithiol compound in the mitochondria found in meat and small amounts of fruits and vegetables that has potential anti-inflammatory, antioxidant, and cognitive benefits [49,50]. A randomized placebo-controlled pilot trial demonstrated that combined omega-3 (675 mg DHA + 975 mg EPA) and LA (600 mg/day) supplementation for 12 months to 39 probable AD subjects 55 years or older yielded cognitive benefits [51]. Their results showed no differences in changes in urine F2-isoprostane (an oxidative stress and inflammatory marker) between groups at 6 months or 12 months. In the cognitive measures, only omega-3 and LA combination therapy decreased the rate of decline in mini-mental state examination, and the other groups showed no difference from that of the placebo group. However, both omega-3 and omega-3 + LA showed less decline in instrumental activities of daily living for the functional measures. All the interventions were well-tolerated with minimal adverse events. This study showed the potential of combining omega-3 and LA to enhance cognitive function in pro-AD subjects, although the mechanism seemed irrelevant to reduced oxidative stress.

Please see Table 1 for summarized information on clinical studies of FO and natural product combinations in improving or protecting cognitive function. 

## 5. Mechanism Studies of FO and Natural Products in Improving Cognition

Since clinical trials supporting the beneficial use of FO and natural ingredient combinations are largely lacking and outcomes remain inconclusive, mechanism studies were sought for potential combinations. Nineteen articles were included in this study that investigated various combinations of FO and natural products, addressing various pathological components occurring in cognitive dysfunction.

### 5.1. Combination Therapies of FO and Natural Products Modulation of Neuronal Development, Function, and Survival

Neuronal development, function, and survival are integral components of the intricate processes that govern cognitive function throughout an individual’s life. In the context of cognitive abilities, these aspects of neuroscience play crucial roles in shaping and maintaining the brain’s capacity for learning, memory, and overall cognitive performance [52]. It is recognized that homocysteine, an amino acid, tends to increase with age due to factors like altered metabolism and vitamin deficiencies. Elevated homocysteine is associated with cognitive decline and a higher risk of neurodegenerative disorders [53]. This is believed to result from vascular damage, inflammation, and oxidative stress in the brain. Maintaining adequate levels of B vitamins, involved in homocysteine metabolism, may help to mitigate these cognitive risks and support brain health, especially in aging individuals. Brain-derived neurotrophic factor (BDNF), a crucial protein for brain health, is associated with neuronal development, function, and survival, influencing synaptic plasticity, which is essential for learning and memory [54,55]. Aging may lead to decreased BDNF levels, impacting cognitive function. Thus, understanding and promoting BDNF activity are vital for maintaining cognitive function, especially in the aging population. Vitamins and nutrients have been associated with promoting the production and function of BDNF, such as vitamin B6 and B12, and are linked to better cognitive outcomes. Below are the studies that attempted to combine FO and the vitamin B group to fight against aging-induced cognitive decline in relation to homocysteine and BDNF.

#### 5.1.1. FO and Curcumin

Curcumin is recognised to reduce neuroinflammation in cognitive disorders by inhibiting the production of proinflammatory cytokines such as interleukin (IL)-1β, IL-6, IL-8, tumour necrosis factor (TNF), and inducible nitric oxide synthase (iNOS) [56,57,58]. Its molecular mechanisms are associated with the suppression of oxidative stress, mitogen-activated protein kinase (MAPK), nuclear factor erythroid 2-related factor 2 (Nrf2), and nuclear factor-kappa B (NF-κB) [59]. Due to its prominent effects in reducing neuroinflammation, curcumin was examined together with FO/DHA for improving cognition in the following studies.

**Figure 2:**
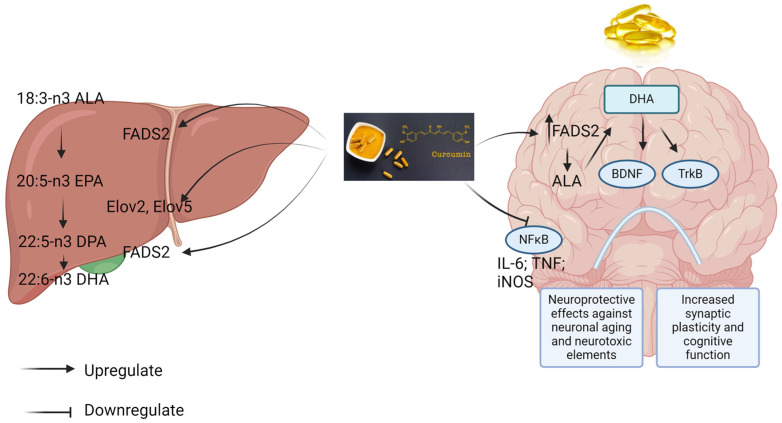
Schematic of the influence of curcumin on mammalian DHA synthesis pathway and its neuroprotective effect [61]. ALA: alpha-linolenic acid; DPA: docosapentaenoic acid; FADS2: fatty acid desaturase 2; Elov: elongase; BDNF: Brain-derived neurotrophic factor; TrkB: tropomyosin receptor kinase B; NF-κB: nuclear factor-kappa B; IL-6: interleukin-6; TNF: tumor necrosis factor; and iNOS: inducible nitric oxide synthase.

An in vivo study from Wu et al. (2014) investigated the combined effects of curcumin- and/or DHA-enriched diets (DHA, 1.2%; Cur, 500 ppm) for 2 weeks in rats with post traumatic brain injury [60]. In cognitive tests performed in a Barnes maze, groups receiving the combination displayed even greater beneficial effects in learning performance than separate applications. The combination showed greater effects in restoring BDNF and phosphorylated-tropomyosin receptor kinase B (p-TrKB) expressions, both critical biomarkers for neuronal survival and growth. The mechanisms underpinning the enhanced effects of the combination were also associated with preserved DHA in the plasma membrane and reduced lipid peroxidation when curcumin was added. Moreover, curcumin was observed to intersect with DHA pathways in the brain, leading to a reduction in the metabolism of DHA [60]. This finding is supported by Wu et al. (2015), in which curcumin increased DHA synthesis from its precursor, alpha-linolenic acid (ALA), by elevating fatty acid desaturase 2 (FADS2) and elongase (Elov) 2 via the fatty acid desaturase 2 (FASD2)-dependent pathway [61]. Interestingly, another study conducted by Sharman et al. (2019) suggested that adding curcumin (0.59 g/kg) to a mixture of DHA (0.36 g/kg), ALA (1 g/kg), and EGCG (0.36 g/kg) did not result in extra benefits in potentiating neuroinflammation and neuroprotective effects compared to the mixture alone in male Tg2576 transgenic mice [62]. The findings from preclinical investigations combining curcumin with FO/DHA appear to be preliminary, possibly due to variations in the study designs and dosages used. A diagram for the proposed interaction between curcumin and the DHA metabolism pathway is shown in Figure 2.

#### 5.1.2. FO and Citicoline

Citicoline, also known as cytidine diphosphate-choline, is a dietary supplement to improve brain performance and memory [63]. Both citicoline and DHA are essential intermediates in the synthesis of phosphatidylcholine.

Nakazaki et al. (2019) investigated the combined effects of DHA and citicoline on improving cognition in ischemic mice [64]. Their results revealed that the oral administration of citicoline (40 mg/kg body weight/day) and DHA (300 mg/kg body weight/day) for 11 days significantly improved the learning and memory ability of ischemic mice, of which the combined effect was greater than that of each treatment alone. In addition, the combination was found to prevent neuronal cell death and slightly increase DHA-containing phosphatidylcholine. This study showed the potential benefits of combining DHA with citicoline for enhanced benefits in improving memory deficits post-ischemic damage.

#### 5.1.3. FO and Vitamin B

The term ‘B vitamins’ refers to a group of eight water-soluble vitamins that have critical roles in the anabolic and catabolic metabolisms. Vitamin B6, consisting of three chemically distinct compounds (pyridoxine, pyridoxal, and pyridoxamine), is crucial for regulating mental function and is involved in the remethylation of homocysteine. Deficiency in Vitamin B6 is believed to elevate homocysteine levels and may lead to conditions such as seizures, migraines, cognitive impairment, AD, and other forms of dementia [65]. Vitamin B12 is essential for the normal functioning of the brain and nervous system, being involved in metabolic processes and serving as a co-factor for myelin formation. Deficiency may lead to severe symptoms and increased risks of cognitive decline, dementia, and AD [66]. However, there is no solid evidence regarding the effectiveness of oral supplements of vitamin B6 or vitamin B12 in improving cognitive functions, as shown by Cochrane reviews [67]. Systematic reviews and meta-analyses revealed that vitamin B supplementation could lower serum homocysteine levels, however, this did not translate into cognitive improvement in elderly patients with AD or dementia [68].

Three in vivo studies investigated the combined effects of n-3 PUFAs and vitamin B12 supplementation on cognitive performance. In a study by Rathod et al. (2015), the beneficial effects of vitamin B12 and omega-3 fatty acids were demonstrated across two generations on brain development and function in pregnant Wistar rats [69]. Subsequently, Rathod et al. (2016) suggested that the combination exhibited the highest protein and gene expressions of nerve growth factor (NGF) in the cortex and hippocampus in the Wistar rats. NGF is essential for neuron neurite outgrowth, survival, and development in the peripheral and central nervous system [70]. This combination also led to greater levels of BDNF in both the hippocampus and cortex and improved cognitive performance in comparison to that of vitamin B12 deficiency, B12 deficiency, and omega-3 fatty acids and vitamin B12 alone [71].

### 5.2. Combination Therapies of FO and Natural Products Targeting Neuroinflammation

Neuroinflammation refers to the inflammatory responses in the brain or spinal cord attributed to the activation of neuroimmune cells microglia and astrocytes into pro-inflammatory states [72,73]. It is being increasingly recognized that neuroinflammation triggers and accelerates the pathophysiology of neurodegenerative disease, such as AD and dementia in older people, playing a central role in the early onset of cognitive decline [74]. Thus, neuroinflammation is deemed as an emerging therapeutic target for various neurodegenerative diseases, including dementia [75].

Increasing studies have demonstrated the capacity of FO to reduce neuroinflammation and improve cognitive function in various disease models, such as streptozotocin-induced diabetic rats [76], postoperative delirium-like behaviour in mice [77], and obese mice [78]. FO regulates neuroinflammation through oxylipins formed during enzymatic processes or auto-oxidation metabolism. Oxylipins serve as precursors for pro-resolving mediators like resolvins and lipoxins, which play anti-inflammatory roles [79]. Four studies investigated the possibility of adding nutraceuticals to FO to potentiate its effects in reducing neuroinflammation and, thus, potentially leading to improved cognitive function.

#### 5.2.1. FO and Resveratrol

Resveratrol (Res) is a polyphenol that belongs to the stilbenes group presented in dark-coloured grapes, mulberries, peanuts, and red wine. It is a popular nutraceutical known for its anti-inflammatory and antioxidant activities [80,81]. The mechanisms of Res in reducing neuroinflammation are associated with the inhibited transcription of pro-inflammatory genes, including NF-κB and activator protein 1 (AP-1) [82].

The animal study by Thomas et al. (2013) investigated the potential therapeutic effects of Res-DHA combination on modulating hippocampal gene expressions in adult C57Bl/6 mice [83]. The animals were grouped and supplemented with either 50 mg/kg/day of Res, DHA, or a combined supplementation of 50 mg/kg/day each for six weeks. When administered in combination, DHA and Res showed a higher capacity to decrease apolipoprotein E (ApoE) expression, known to induce Aβ production and oxidative stress in neurodegenerative mechanisms, whereas individual Res or DHA did not alter ApoE expressions. The gene expressions of antioxidants such as superoxide dismutase 1 (SOD1), superoxide dismutase (SOD2), and heme oxygenase 1 (Hmox1) were increased with the combination, which was not seen in DHA or Res alone. Interestingly, the modulatory effect on neuro-inflammatory-related genes differed for each individual and combined supplement [83]. The pathway analysis based on the modulated genes in each group demonstrated that the janus kinases (JAK) signal transducer and activator of the transcription (STAT) signalling pathway was particularly enriched by both individual and combined treatment. A genome-wide association (GWAS) study investigated the overlapping gene targets and signalling pathway between EPA/DHA and resveratrol. Their results revealed that EPA/DHA and resveratrol exhibited commonality in an enrichment of pathways associated with immune responses, disease signalling, cardiovascular function, and signal transduction across all treatment conditions [84].

#### 5.2.2. FO and Vitamin A and D

An in vitro study investigated the potential of combining vitamin A and D with DHA and EPA to reduce neuroinflammation in lipopolysaccharides (LPS)-induced microglia BV2 cells [85]. Their results showed that individual DHA (20 µM), vitamin A (0.583 or 1.75 µM), and D (0.1 or 1 µg/mL) reduced nitric oxide (NO) and IL-6 to various extents, with significance compared to LPS stimulation. However, EPA (20 µM) did not exhibit significant reductions. Combining the four ingredients showed greater effects in suppressing NO and IL-6 compared to each individual component [85]. It is noted that a comparison between the combined group and individual component was only made at a certain dosage level.

#### 5.2.3. FO and Polyphenols

An in vitro study aimed to assess the effectiveness of EPA and multiple phenolic compounds, including lyc-O-mato^®^, carnosic acid, and lutein, in reducing neuroinflammation in LPS-induced microglia BV2 cells. Their results suggested that the addition of 0.1 µM Lyc-O-mato^®^/0.2 µM carnosic acid/0.2 µM lutein to EPA (0.03 to 2 µM) led to a greater inhibition of NO production than using EPA alone. Notably, the combination of 0.1 µM Lyc-O-mato^®^, 0.2 µM carnosic acid, and EPA showed the highest inhibitory effects among all tested mixtures in reducing LPS/interferon (IFN)-γ-induced NO, prostaglandin E2 (PGE2), and IL-6. The mechanisms were associated with down-regulated p-NFκB and cyclic adenosine monophosphate (cAMP) response element-binding protein activation, both key transcription factors in inflammation and neuronal plasticity, learning, memory, and cell survival [86].

#### 5.2.4. FO and Lipoic Acid

Lipoic acid (LA) possesses strong antioxidant properties and has the ability to regenerate other antioxidants like vitamin C, vitamin E, and glutathione [87]. Thus, studies attempted to combine FO and LA to combat oxidative stress and neuroinflammation to improve cognitive function.

An in vivo study investigated the combined effects of FO and LA on protecting cognition in sepsis rats [88]. Individual or combined FO (Omegaven 10%) and LA (1 mL/200 mg/kg) were administered to Wistar rats daily through oral gavage immediately after surgery, at 12 h after surgery, and at 24 h after surgery. Sepsis in the Wistar rats was induced by cecal ligation and perforation. The combination showed the most prominent effects in reducing TNF and IL-1β in the cortex, prefrontal cortex, and hippocampus. This finding is consistent with the anti-cytokine effects of the combination on LPS-stimulated microglia cells. FO-LA also significantly restored the expressions of BDNF in the cortex and hippocampus, which were greater than those in the individual group. Subsequently, behaviour impairment was not observed in the FO-LA group.

### 5.3. Combination Therapies of FO and Natural Products Targeting Oxidative Stress

The brain is particularly sensitive to oxidative stress due to its large oxygen consumption and high lipid content [89]. During oxidative phosphorylation in the mitochondria, free radicals, specifically oxygen and nitrogen species, are generated. The heightened reactivity resulting from unpaired electrons contributes to elevated oxidative stress. Accumulative evidence suggests that oxidative stress is involved in the onset and progression of aging-induced cognitive decline and AD, as free radical damage to mitochondria can lead to an increased production of toxic amyloid beta [90]. During the aging process, oxidative damage to nuclear and mitochondrial DNA occurs with diminished repair mechanisms. Consequently, oxidative stress is intricately connected to age-related damage, affecting learning and memory [91].

A double-blind RCT investigated the effectiveness of daily FO (0.45 g EPA and 1 g DHA) on AD patients for 12 months. The results demonstrated that FO administration significantly decreased oxidative stress markers and improved the membrane fluidity in plasma [92]. However, n-3 PUFAs are vulnerable to lipid peroxidation. When the levels of reactive oxygen species (ROS) are excessively high and antioxidant defences are compromised, lipid peroxidation can occur, impacting the stability of DHA and EPA [93]. Thus, it is hypothesized that composite antioxidants agents can improve the oxidative stability of n-3 PUFAs, potentially leading to an advanced outcome.

#### 5.3.1. FO and Epigallocatechin-3-Gallate

Epigallocatechin-3-gallate (EGCG) is a polyphenolic flavonoid belonging to the catechin subgroup of flavonoids, which are potent antioxidants largely found in *Camellia sinensis* and *Camellia assumica* [94,95]. The radical scavenging ability of EGCG is largely attributed to the dihydroxyl groups on its B and D rings and the critical galloyl moiety, which consists of three phenolic and carboxyl groups [96,97]. EGCG was found to exhibit anti-amyloidogenic effects attributed to the inhibited aggregation of α-synuclein and amyloid-β protein expression, which led to the deposition of pathological amyloid proteins [98]. Several preclinical studies have investigated the plausible beneficial effects of combining FO/DHA/EPA with EGCG on improving cognition by combating oxidative stress.

The combination of FO (8 mg/kg/day) and EGCG (62.5 mg/kg/day or 12.5 mg/kg/day) markedly reduced Aβ deposition in Tg2657 mice, whereas EGCG alone did not lead to any significant effect [99]. Interestingly, higher plasma free EGCG in the mice was measured in the combination group compared to EGCG alone, which could potentially lead to a higher ECGC concentration that freely penetrates the blood–brain barrier (BBB). Sharman et al. (2019) also demonstrated the prominent effects of EGCG, DHA, and ALA combined in a certain ratio on decreasing the amyloid plaque load [62]. An in vitro study investigated the interaction of EGCG and DHA on oxidative damage in FaO cells, a subclone of the H4-11-E-C3 rat hepatoma. Their results suggested that EGCG-DHA (molar ratio 4:1, total dosage 50 µM) rescued cells from tert-BHP-induced cytotoxicity, whereas DHA alone was not able to. The effects of EGCG-DHA appeared to be greater than those of EGCG alone. Moreover, DHA seemed to induce a higher ROS production and reduce antioxidant defences, whereas EGCG or adding EGCG to DHA reversed this change [100].

#### 5.3.2. FO and Quercetin

Quercetin is a polyphenolic natural product with noted anti-inflammatory, antioxidant, and anti-microbial actions, which is commonly found in fruits and vegetables such as apples, oranges, and kale [101,102]. Due to the catechol and hydroxyl groups in its B and AC rings respectively, quercetin has the optimal structure for the scavenging of free radicals, and is, thus, one of the most potent antioxidants in the flavonoid class [103]. Quercetin is also noted to have biological impacts on neuro-cognitive performance [102].

Investigations of the therapeutic potential of FO and quercetin combination therapy for neuroprotection have been conducted in in vivo studies. Joseph et al. (2012) investigated the effects of a 14-day treatment of individual or combined FO [2 mL/kg body weight (BW)] and quercetin (25 mg/kg BW) on 3-nitropropinioc-acid induced oxidative stress in rat brain [104]. Their results demonstrated that the complete normalization of oxidative markers, including ROS, malondialdehyde, protein carbonyls, and NO, was only shown in the combination group, compared to other individual groups. The reduced oxidative stress resulted in a higher degree of neuroprotection, although the mechanisms of this were not fully explored. Subsequently, the same authors investigated this combination in chronic rotenone-induced Parkinson’s disease (PD) rats for 21 days [105]. The results also showed superior neuroprotective activity for the combination against PD-induced cognitive decline. The mechanisms were associated with reduced oxidative stress, lipid peroxidation, and improved antioxidant enzymes. It is speculated that FO and quercetin may target multiple cascades in ROT-induced oxidative stress and neurotoxicity, however, the mechanisms of this remain unclear.

#### 5.3.3. FO and Ferulic Acid

Ferulic acid (FA) is a polyphenolic natural compound with anti-carcinogenic, anti-inflammatory, and antioxidant effects found commonly in oranges, tomatoes, sweet corn, and carrots [106]. The strong antioxidant activity displayed by FA is attributed to the phenolic nucleus and unsaturated hydrocarbon side chain, which allows the compound to form a phenoxy radical through the loss of a hydrogen atom [107]. The phenoxy radical is highly resonance stabilised and is unable to initiate or maintain a chain reaction, resulting in the effective minimisation of a radical chain reaction [107].

Denny Joseph and Muralidhara (2014) investigated the combination of FO (2 mL/kg BW) and FA 50 mg/kg BW) for 2 weeks on 3-nitropropionic-acid-induced oxidative stress and neurotoxicity in rats [108]. The combination demonstrated greater effects on protecting neurons compared to each individual group, as evidenced by restored balance and motor coordination, normalised oxidative markers, and increased antioxidant enzymes.

#### 5.3.4. FO and Astaxanthin

Astaxanthin (AX) is a carotenoid xanthophyll, a red pigment with multiple biological effects including anti-inflammatory, antioxidant, and purported anti-cancer activity, which is derived from marine microorganisms and animals [109]. As a potent antioxidant, AX contains a long polyene chain with double bonds that can bind to monoatomic oxygen species and other radicals to negate oxidative stress [110]. Furthermore, AX is believed to prevent lipid peroxidation through its unique interaction with the cell membrane, and, in essence, assist in the removal of free radicals from the inside to the outside of the cell [111].

Polotow et al. (2015) explored the combination of FO rich in n-3 PUFAs (17 mg EPA + DHA/kg/day) and astaxanthin (1 mg/kg BW) five days/week for 45 days on the redox status and neuroinflammation indexes in the cerebellum and motor cortex of Wistar rats [112]. In addition, krill oil (10 mg EPA/kg BW + 4.7 mg DHA/kg BW + 0.0072 mg ASTA/kg BW) was supplied, as it naturally contains both nutraceutical ingredients. Surprisingly, the individual treatment of ASTA exhibited significant changes in the redox metabolism, whereas krill oil or FO-AX did not alter the redox or inflammatory indices in neuromotor-associated rat brain regions. Another two in vivo studies suggested that the daily (45 days) intake of FO (10 mg EPA/kg BW and 7 mg DHA/kg BW) and/or natural ASTA (1 mg ASTA/kg BW) improved the redox balance in the plasma and supressed activated T- and B-lymphocytes proliferation in the Wistar rats [113,114]. Thus, there remains a potential of combined FO-AX to exhibit additional benefits either through their interaction in the plasma or after penetrating the BBB.

See Table 2 for a summarised list of FO combinations with natural products in addressing various pathological components in cognitive decline.

## 6. Discussion

### 6.1. Major Findings

Cognitive decline is a common condition among older people, and there is currently a lack of viable treatment for its prevention or management. Fish oil (FO) contains a rich amount of n-3 fatty acids, including DHA and EPA, which are often recommended as beneficial dietary supplements for protecting cognitive function. However, the current evidence from clinical trials to support its use remains controversial. Furthermore, the utilization of FO on a global scale is not aligned with sustainable development principles. As a result, various efforts have been made to explore alternative sources and address this sustainability concern. Combination therapy that adds FO to natural products may be capable of addressing the multimodal pathological components occurring in cognitive decline, which has become a promising pharmacological strategy. This approach may provide a solution with a reduced amount of FO and improved efficacy in protecting cognitive function. This study reviewed combinations of FO and natural products that claim advanced effects in addressing various cognitive risk factors and related mechanisms.

The clinical evidence presents a mixed narrative for combination therapies, with a placebo-controlled RCT indicating a positive impact on cognitive performance with omega-3 (675 mg DHA + 975 mg EPA) and LA (600 mg/day) supplementation for 12 months. However, other trials suggest that the effectiveness of FO/omega-3 with curcumin does not yield additional health benefits. This negative outcome might be contingent upon factors such as dosage and the specific composition employed. In addition, combinations of FO–vitamin E result in enhancements in short-term memory, though overall cognitive factor scores remain largely unaffected. This prompts a call for further investigation to elucidate the intricate interplay between FO and these nutraceuticals in influencing cognitive outcomes in preclinical studies. Notably, supplementation with the Vitamin B group with a high serum DHA level demonstrates potential benefits in cognitive outcomes, although no RCTs have been conducted to investigate the clinical outcomes of co-administration.

A number of preclinical studies have explored combining FO with natural products to bolster neuronal development, function, and survival. Addressing the common thread of neuroinflammation in neurodegenerative diseases, combination therapies involving FO and natural products like curcumin, resveratrol, and polyphenols show promise in reducing inflammation and improving cognitive function. Likewise, oxidative stress, implicated in cognitive decline and AD, becomes a focal point. The combination of FO with antioxidants such as EGCG, quercetin, lipoic acid, ferulic acid, and astaxanthin emerges as a potential avenue for mitigating oxidative stress and enhancing cognitive function. These studies can provide informed decisions for the research design and target population in future clinical trials.

### 6.2. Limitation and Knowledge Gap

Overall, while studies present promising results for combining FO with various natural products to address cognitive decline in preclinical studies, none of these combinations present solid evidence of advanced effects in addressing certain pathological components in relation to cognitive decline with coherent preclinical studies that is ready to be utilised in clinics or for industry manufacturing. This lack of conclusive findings in clinical trials, although results from preclinical research were promising, may reflect the lack of knowledge on optimal compositions for the combination, bioavailability, limited characterisation of possible interactions, and insufficient trial duration. Basic research is essential to establish the optimal dosages, compositions, and mechanisms of action for these combinations to be tested in clinical trials. The complex interplay of factors influencing cognitive health requires a comprehensive and nuanced approach when designing effective therapeutic strategies.

Assessing the efficacy of FO and natural products in combinations has proven challenging. There is a shortage of cellular and animal studies dedicated to evaluating the plausible synergistic effects of combinations before progressing to clinical trials. Although many studies have reported advanced or synergistic effects of FO and natural products on addressing various aspects of pathogenesis of cognitive decline, rigorous mathematical methods (such as combination index or isobologram) are lacking to assess the interactions in these combinations to substantiate the claim of synergy. Thus, solid evidence to support each combination with synergistic effects remains lacking. Nowadays, there is rapid development and evolution in using artificial intelligence machine learning to assess and predict the interactions in complex combinations, as well as simulate the dose-response effects. These techniques could be used to thoroughly assess the plausible synergy occurring in combinations with FO. In addition, network pharmacology has been widely applied to elucidate the molecular targets of complex combinations in addressing certain pathological conditions, including cognitive decline, neuroinflammation, and oxidative stress. This technique can facilitate the investigation of the molecular mechanisms underpinning the development of FO combination therapy and guide experimental studies for further investigation.

It was noticed that most of the studies included in this review only examined the pharmacodynamic interactions of FO combinations. Whether there were any interactions, occurring in terms of bioavailability in the plasma or cerebrum, remains under explored. Interestingly, several studies mentioned the possibility of such interactions of preserved or enhanced concentrations when adding another component, but further study is needed to elucidate such a mechanism. For example, higher plasma free EGCG was found when used together with FO compared with EGCG alone, which can potentially lead to higher concentrations freely penetrating the BBB and enhanced effects in reducing oxidative stress. A few clinical trials reported the correlation of the plasma DHA level with the cognitive protective effect of vitamin B. These clinical trials recommended searching for a suitable cohort for examining the benefits of vitamin B treatment, however, this also highlights a novel mechanism of the possible crosstalk of vitamin B and DHA, which warrants further studies.

Given the vast amount of studies on FO in protecting cognitive function, it remains challenging to understand the individual roles of DHA and EPA and their crosstalk in the context of cognitive function. Thus, this varied when developing the combinations across the studies where either EPA, DHA, omega-3 fatty acids, or FO were used. This reflects the lack of understanding of the functional role of each component and the mechanisms of the possible interaction with other natural products. While most of the studies focused on FO or DHA, the impact of EPA on the interaction pathway with or without the addition of other components lacks study. This again needs rigorous preclinical study to lay the foundation and rationale for the design of an optimal combination.

### 6.3. Recommendations

In light of the multifaceted findings emerging from studies that delved into the combination of FO/DHA/EPA with diverse nutraceuticals to address cognitive decline, it appears that the development of FO combination therapies in protecting cognition remains in an early stage. Several considerations and potential recommendations can be discerned for navigating the option of FO combination therapies for the realm of cognitive health before their wide applications.

When examining the clinical outcomes for FO–vitamin B combinations, it was noticed that the baseline DHA level in the cohort is closely linked with the clinical outcomes. Recognising the variability in outcomes across different cohorts underscores the imperative for personalized approaches in strategies or that addressing the suitable population for the treatment served a more rigorous design of the clinical trials. Thus, the complexity of cognitive health necessitates a holistic assessment of patients, incorporating factors such as baseline omega-3 fatty acid levels, existing cognitive health status, and the presence of any coexisting conditions. This comprehensive evaluation serves as a foundation for more targeted and effective therapeutic recommendations. Most of the clinical studies incorporated in this review only examined subjects with limited timepoint. As cognitive decline is a chronic condition, the regular monitoring of cognitive outcomes emerges as a key practice, with clinicians advised to track changes in cognitive function, memory, and other relevant parameters. This ongoing assessment is integral to gauging the effectiveness of the prescribed combined interventions.

In addition, a strong foundation of preclinical studies is necessary to identify the suitable pathological targets and associated molecular targets that underpin the advanced effects of a combination. FO and n-3 PUFAs are mixtures of compounds, per se, and exhibit multi-target actions. Most of the combinations were designed in a way that the added natural products addressed one or more of the molecular targets that FO was addressing, and, thus, it was expected to exert a strengthened effect with the combination. However, current studies do not examine the optimal ratio of the combination, i.e., which component in n-3 PUFAs would be the key component underpinning the advanced effects of the combination and associated molecular targets. This could be attributed to the unsatisfying outcome when moving to clinical trials. In addition, the design of most of the preclinical and clinical trials lacked coherence. Thus, it is essential to obtain a comprehensive understanding of the optimal dosages and compositions for combined interventions, as well as the molecular mechanisms and suitable pathological targets. A strong rationale is necessary before subjecting a combination to clinical trials for further development.

Neuroinflammation and oxidative stress are the two major pathological components driving the progress of cognitive decline. Numerous studies were found to investigate the combination of FO and natural products (i.e., resveratrol, EGCG, lipoic acid, and quercetin), which showed promising preliminary results in improving cognition by addressing these two main pathological pathways. While further studies are warranted to explore the enhanced efficacy of each combination, serum biomarkers could be included in overall cognitive health management plans for suitable patients. Further to this integration with existing therapeutic strategies, a holistic and well-coordinated approach will be considered for cognition well-being.

For exploring novel combinatory interventions with FO, a number of studies were found to investigate the combined effects of FO and phosphatidylserine in cognition and ADHD-related memory and attention symptoms with promising outcomes. However, none of the studies assessed the combined effects in comparison with the individual effect, and thus, if there were advanced effects in the combination cannot be determined. In addition, medicinal mushrooms are considered to serve as a primary ingredient alongside FO to enhance cognitive function. Specifically, *Hericium erinaceus* stands out as a well-established candidate among various culinary medicinal mushrooms for promoting a “healthy brain” [115]. The potential of *H. erinaceus* to improve cognitive function has been demonstrated in elderly cohorts with mild AD or MCI [116,117,118], although the molecular mechanisms underlying this effect have not been fully elucidated.

Nevertheless, as the landscape of FO-related cognitive health research evolves rapidly, the continuous updating of knowledge enables advanced knowledge on the complex domain of cognitive health and ongoing research of FO therapies for their sustainable development.

## 7. Conclusions

In conclusion, this review summarizes the combined effects of FO and natural products on improving cognitive function by protecting cerebral function, recognition, and memory, reducing neuroinflammation and oxidative stress. While some evidence suggests a synergistic effect between FO and natural products, conclusive proof remains elusive. Consequently, future research may focus on elucidating the optimal composition, assessing interactions, identifying suitable cohorts and disease targets, investigating molecular mechanisms, and determining the appropriate treatment durations. The findings reported in this review, however, portray plausible developments for pharmacological combinations against cognitive decline that support the sustainable development of FO.

## Figures and Tables

**Table 1:** Clinical studies of FO and natural product combinations in improving or protecting cognitive function.

FO/DHA/EPA and Dosages	Natural Products and Dosages	Type of Study	Target Population	Treatment Duration	Key Results	References
FO (2000 mg/d DHA + 400 mg/d EPA, Blackmores Omega Brain^TM^)	Curcumin (160 mg/d, Blackmores Brain Active^TM^)	Double-blind RCT	152 older sedentary overweight/obese adults	16 weeks	Individual treatment of FO or curcumin improved cerebrovascular responsiveness vs. placebo group in males only; Curcumin + FO did not generate any further improvement in cognition	[38,39]
FO (480 mg EPA + 480 mg DHA)	Vitamin E (50 IU)	Double-blind RCT	160 healthy male and female volunteers aged 50 to 70 years	16 weeks	No improvement in cognitive factor scores in individual or combined treatment; A significant time by treatment effect for short-term memory following 6 weeks of treatments	[45]
High plasma omega-3 fatty acid concentrations (>590 μmol/L)	Vitamin B supplementation (0.8 mg folic acid, 0.5 mg vitamin B12 and 20 mg vitamin B6)	A retrospective analysis	168 elderly people with MCI	2 years	B vitamin group lowered the mean atrophy rate by 40% in subjects with high plasma omega-3 fatty acid concentrations compared with that of placebo and the subjects with low baseline omega-3 fatty acids and homocysteine levels	[46]
High baseline omega-3 fatty acids (515.98 ± 235.01 serum DHA + EPA)	High does B vitamins (composition not specified)	Placebo-controlled RCT	266 elderly individuals with MCI	2 years	33% of individuals were prone to developing dementia, as assessed by clinical dementia rating scale, compared to 59% of those on placebo; The interaction appears to occur between B vitamins and DHA only, with EPA demonstrating a comparatively lower impact	[47]
High baseline omega-3 fatty acid levels with DHA at around 4.3 ± 1.2%	Vitamin B (500 µg V12 and 400 µg folic acid)	A post hoc analysis study within the B-proof trial, a randomized, double-blind placebo-controlled trial	191 elderly individuals aged over 65 years old with elevated homocysteine levels	2 years	Participants benefiting significantly more in improving global cognition from B-vitamin if they were also in the high DHA tertile; however, such trend was not observed in measuring domain-specific cognitive functioning	[48]
Omega-3 (675 mg DHA + 975 mg EPA)	LA (600 mg/day)	Randomized placebo-controlled pilot trial	39 probable AD subjects with 55 years or older	12 months	Only combined therapy decreased the rate of decline in mini-mental state examination; Both omega-3 and omega-3 + LA showed less decline in instrumental activities of daily living for the functional measures; All the interventions were well-tolerated with minimal adverse events	[51]

**Table 2:** Preclinical studies of FO and natural product combinations with determined or indicated synergistic activities in improving or protecting cognitive function.

FO/DHA/EPA	Natural Products	Type of Study and Subjects	Key Results	Mechanism of Synergy or Enhanced Activity	Method of Synergy Determination
DHA-enriched diets (DHA, 1.2%) for 14 days [60]	Cur (500 ppm)	In vivo: rats with post traumatic brain injury	Combination exhibited an even greater beneficial effects in learning performance than separate applications	Improved neuronal survival and growth (BDNF and p-Trk) Preserved DHA in the plasma membrane and reduced lipid peroxidation; Reduced DHA metabolism	Analysis of variance (ANOVA) group comparison
ALA diet (ALA; 2.7% of fat) for 3.5 weeks [61]	Cur (500 ppm)	In vivo: male Sprague–Dawley rats	Curcumin complements the action of DHA in the brain	Increased DHA synthesis by curcumin, leading to elevated brain DHA content	One-way ANOVA followed by Tukey’s post hoc test for multiple comparisons
DHA (0.36 g/kg), ALA (1 g/kg) for 12 months [62]	EGCG (0.36 g/kg) and curcumin (0.59 g/kg)	In vivo: male Tg2576 transgenic mice	The combination did not result in extra benefits in potentiating neuroinflammation and neuroprotective effects	Not applicable	One-way ANOVA for group comparison
DHA (300 mg/kg body weight/day) for 11 days [64]	Citicoline (40 mg/kg body weight/day) for 11 days	In vivo: ischemic mice	Combination significantly improved learning and memory ability with an effect greater than individual treatment	Prevent neuronal cell death and slightly increased DHA-containing phosphatidylcholine	ANOVA followed by multi-group comparisons
Combination of DHA (120 mg) and EPA (180 mg) per capsule [69]	Vitamin B12 (25–50 μg/kg diet)	In vivo: pregnant Wistar rats and second generation	Combination showed significantly higher cognitive performance	Further enhanced DHA and BDNF in the hippocampus and cAMP response element-binding protein (CREB) mRNA	One-way ANOVA followed by Tukey’s honestly significant difference as post hoc test
Combination of DHA (120 mg) and EPA (180 mg) per capsule [70]	Vitamin B12 (25–50 μg/kg diet)	In vivo: pregnant Wistar rats and second generation	Combination improved neurovascular function	Combination exhibited the highest protein and gene expressions of NGF in the cortex and hippocampus	One-way ANOVA followed by Tukey’s post hoc test
Combination of DHA (120 mg) and EPA (180 mg) per capsule [71]	Vitamin B12 (25–50 μg/kg diet)	In vivo: pregnant Wistar rats and second generation	Improved brain development	Greater levels of BDNF in both hippocampus and cortex	One-way ANOVA followed by Tukey’s post hoc test
DHA 50 mg/kg/day each for six weeks [83]	50 mg/kg/day of Res for 6 weeks	In vivo: adult C57Bl/6 mice	Higher capacity in decreasing apolipoprotein E (ApoE) expression	Increased antioxidant gene expressions; enriched JAK signal transducer and STAT signalling pathways	One-way ANOVA followed by Tukey’s post hoc test
DHA (20 µM), EPA (20 µM) [85]	Vitamin A (0.583 or 1.75 µM) or D (0.1 or 1 µg/mL)	In vitro: LPS-induced microglia BV2 cells	Four-ingredient combination exhibited greater anti-neuroinflammatory effects	Further supressed NO and IL-6 expressions	Kruskal–Wallis H test; Mann–Whitney U test with Bonferroni–Holm post-hoc test
EPA (0.03 to 2 µM) [86]	0.1 µM Lyc-O-mato^®^/0.2 µM carnosic acid/0.2 µM lutein	In vitro: LPS-induced microglia BV2 cells	Reduced neuroinflammation with lowered NO, PGE2, and IL-6 expressions	Down-regulated p-NFκB and CREB protein activation	ANOVA followed by multiple comparisons Bonferroni post hoc correction
FO (8 mg/kg/day) for 8 months [99]	EGCG (62.5 mg/kg/day or 12.5 mg/kg/day) for 8 months	In vivo: Tg2657 mice In vitro: N2a cells	Combination markedly reduced Aβ deposition	Enhanced sAPP-α production; FO enhanced bioavailability of EGCG versus EGCG treatment alone	ANOVA, followed by post hoc Bonferonni’s comparison
DHA (10 μM) [100]	EGCG (40 μM)	In vitro: FaO cells	Combination rescued cells from tert-BHP-induced cytotoxicity	Adding EGCG to DHA supressed elevated ROS expression induced by DHA alone and enhanced antioxidant defence	One-way ANOVA followed by the Tukey post-hoc test
FO [2 mL/kg body weight (BW)] for 14 days [104]	Quercetin (25 mg/kg BW) for 14 days	In vivo: 3-nitropropinioc acid induced oxidative stress in rats	Higher degree of neuroprotection in combination compared to individual treatment	A complete normalization of oxidative markers including ROS, malondialdehyde, protein carbonyls, and NO	One-way ANOVA followed by a post hoc Tukey test
FO [2 mL/kg body weight (BW)] for 21 days [105]	Quercetin (25 mg/kg BW) for 21 days	In vivo: chronic rotenone-induced Parkinson’s disease rats	Combination showed a superior neuroprotective activity	Reduced oxidative stress, lipid peroxidation, and improved antioxidant enzymes	One-way ANOVA followed by a post hoc Tukey test
FO (Omegaven 10%) twice after surgery [88]	LA (1 mL/200 mg/kg) twice after surgery	In vivo: sepsis rats	Prevented behaviour impairment and restored the expression of BDNF in cortex and hippocampus	Reduced TNF, IL-1β in the cortex, prefrontal cortex, and hippocampus	One-way ANOVA followed by a post hoc Tukey test
FO (2 mL/kg BW) for 2 weeks [108]	FA (50 mg/kg BW) for 2 weeks	In vivo: 3-nitropropionic acid-induced oxidative stress and neurotoxicity in rats	Combination exhibited higher effects in protecting neuron compared to each individual group; restored balance and motor coordination	Normalised oxidative markers and increased antioxidant enzymes and cytosolic calcium	One-way ANOVA followed by a post hoc Tukey test
FO rich in n-3 PUFAs (17 mg EPA + DHA/kg/day) five days/week for 45 days [112]	Astaxanthin (1 mg/kg BW) five days/week for 45 days	In vivo: Wistar rats	ASTA alone regulated redox metabolism	Enhanced antioxidant properties with a putative mitochondrial-centred action in rat brain	One-tailed t-Student’s test followed by Tukey’s post-test
Daily intake of FO (10 mg EPA/kg BW and 7 mg DHA/kg BW) for 45 days [113,114]	Natural ASTA (1 mg ASTA/kg BW) for 45 days	In vivo: Wistar rats	Improved redox balance in plasma, supressed activated T- and B-lymphocytes proliferation	Increased glutathione-dependent reducing power, increased activity of the glutathione-recycling enzymes glutathione peroxidase and glutathione reductase in non-activated neutrophils, lowered hydrogen peroxide and NO in phorbol 12-myristate 13-acetate-activated neutrophils	One-way ANOVA followed by Tukey’s post-test
